# Characterization of a recently detected halogenated aminorex derivative: *para*-fluoro-4-methylaminorex (4′F-4-MAR)

**DOI:** 10.1038/s41598-019-44830-y

**Published:** 2019-06-05

**Authors:** D. Fabregat-Safont, X. Carbón, M. Ventura, I. Fornís, F. Hernández, M. Ibáñez

**Affiliations:** 1 Research Institute for Pesticides and Water, University Jaume I, Avda. Sos Baynat s/n, 12071 Castelló de la Plana, Spain; 2 Energy Control (Asociación Bienestar y Desarrollo), c/Independencia 384, 08041 Barcelona, Spain

**Keywords:** Drug regulation, High-throughput screening

## Abstract

Despite the fact that 33% of the new psychoactive substances seized in 2015 were synthetic cathinones, the number of these derivatives has been decreasing in the last years, probably as a consequence of the unfavourable effects reported by users. Thus, the list of possible cathinone analogues is expected to get shorter, and it is likely that the same moiety changes applied for the preparation of synthetic cathinones will be applied in the near future to other stimulants in the search for favourable alternatives to controlled substances. This is evidenced by the increase in newly reported substances belonging to stimulant classes other than cathinones. One of the possible candidates for a new backbone from which to base new stimulants is aminorex, which is classified as a Schedule I substance by the Drug Enforcement Administration. Three derivatives have been reported until now: 4-methylaminorex or 4-MAR (also categorized as a Schedule I substance), para-methyl-4-methylaminorex (4,4′-DMAR) and 3′,4′-methylenedioxy-4-methylaminorex (MDMAR). Recently, the new halogenated 4-MAR derivative, para-fluoro-4-methylaminorex, characterised in this work (and abbreviated as pF-4-methylaminorex or 4′F-4-MAR) was detected by the Slovenian police. In the present work, 4′F-4-MAR has been characterized by high resolution mass spectrometry and nuclear magnetic resonance in a sample obtained from an anonymous consumer. This research shows that the same modifications applied for the preparation of synthetic cathinones are being used to prepare new stimulants based on the aminorex backbone.

## Introduction

The use of synthetic stimulants has increased in Europe in the last years, based on the seizures reported by the European Monitoring Centre for Drug and Drug Addiction (EMCDDA) in its 2017 Report^1^. According to this report, 33% of the substances seized in 2015 were synthetic cathinones, and 6% phenethylamines^1^. Synthetic cathinones are, nowadays, the second largest group of new psychoactive substances (NPS) monitored by the EMCDDA, and the most frequently seized NPS in 2015^1^. In the last years, the characterization of novel synthetic cathinones has been an on-going topic for forensic laboratories, showing that modifications of the cathinone moieties are the most frequent option for the production of novel stimulants^2–9^. Nonetheless, the number of new synthetic cathinones reported every year is decreasing^1^, and the effects profile of the newest cathinone derivatives as reported by users have been unfavourable (especially when compared to older cathinone analogues)^10^. While the cathinone backbone appears to be capable of tolerating highly bulky as well as polar substitutions and additions while retaining psychoactivity, it seems that the list of possible analogues is getting shorter. Based on this trend, it is expected that in the next years, these modifications will be keep being applied to other stimulant classes in the search for favourable (to users) alternatives to controlled substances. This is evidenced by the increase in newly reported substances belonging to stimulant classes like the phenethylamines and piperazines^1^.

**Figure 1:**
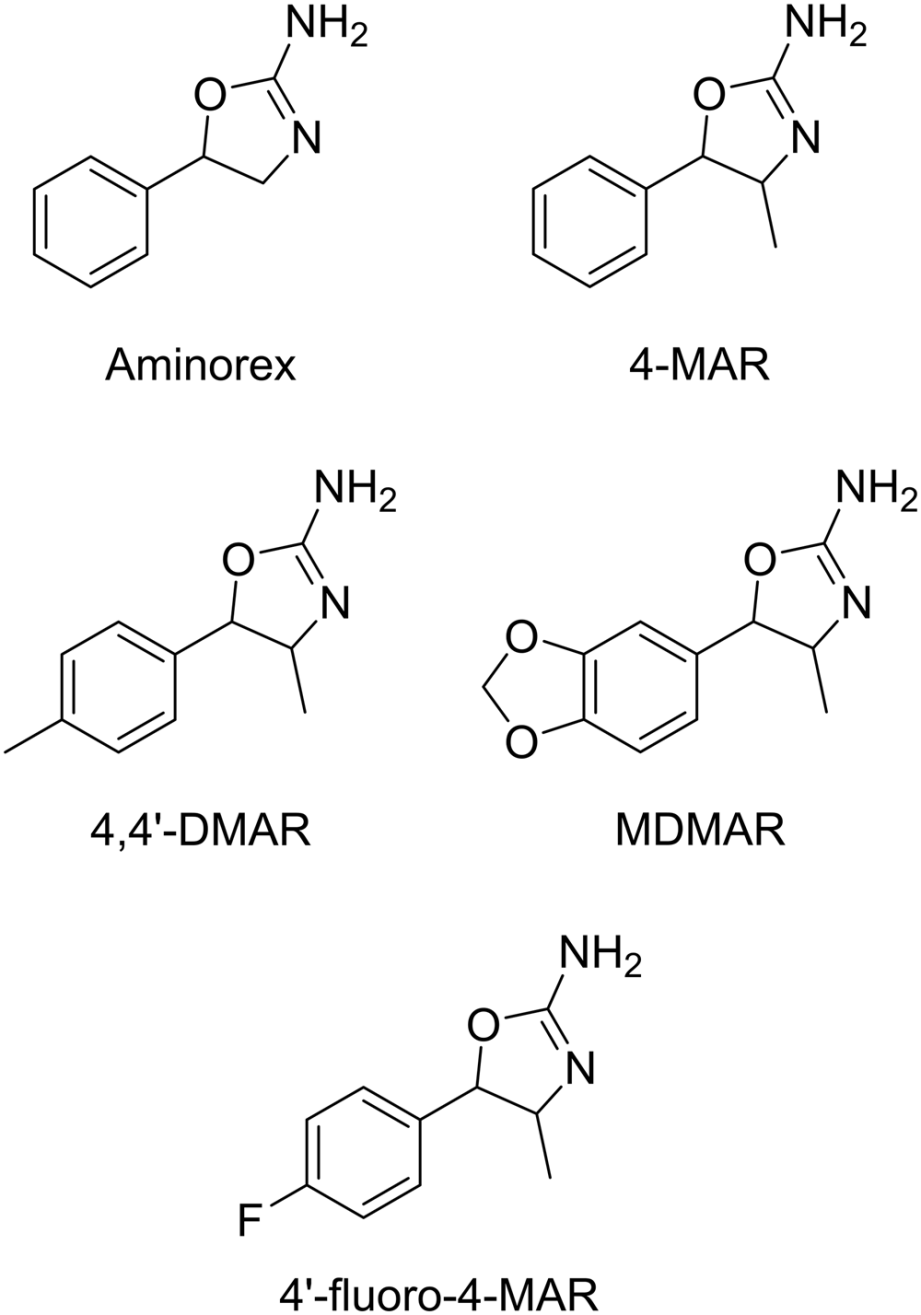
Structure of aminorex and its reported derivatives, including the new derivative reported in this work.

Over the past few years, the cathinone family has been the most common starting point for the production of analogs that belong in the stimulants class^11^. Several other substance families have been used, in much lesser instances, as a backbone to produce analogs, such as amphetamine (2-FA, 3-FA, 4-FA)^12^, phenmetrazine (3-FPM)^13^, methylphenidate^14^, etc. There are not many cases analogs of aminorex^15^, so it remains a possible choice for producers of NPS to explore. Aminorex (Fig. 1) was patented by McNeil Laboratories in 1964 (US Patent 3,161,650) as a new central nervous system stimulant^16^. Aminorex was used in different countries as an appetite suppressor^17^, but was rapidly withdrawn due to the pulmonary hypertension it produced, which was linked with several deaths^18,19^. In the United States, aminorex is classified as a Schedule I substance by the Drug Enforcement Administration (DEA)^20^. The most well-known aminorex derivative is 4-methylaminorex or 4-MAR (Fig. 1), also patented by McNeil Laboratories in 1966^21^. Several years ago, it was discovered that 4-MAR produces stimulant effects similar to those of cocaine^22–24^, as well as several neurochemical effects complications derived from its use^25^, leading to a fatality related to this compound^26^. For this reason, 4-MAR is also categorized as a Schedule I substance in the U.S.^20^.

In 2014, para-methyl-4-methylaminorex (4,4′-DMAR) (Fig. 1), a novel 4-MAR derivative, was reported and characterized^15,27^. Together with Europol, EMCDDA published an early warning notification in 2014, notifying of the involvement of 4,4′-DMAR in several deaths in Europe, 8 of them in Hungary (June 2013) and 18 in the United Kingdom (between June and December 2013)^28^. Additionally, EMCDDA-Europol published a joint report providing all the information available for 4,4′-DMAR^29^. One year later, the European Union decided to apply control measures to this aminorex derivative^30^. Nevertheless, in 2014 another 4-MAR derivative was reported and characterized, the 3′,4′-methylenedioxy-4-methylaminorex or MDMAR (Fig. 1)^31^. At the time of writing this paper, no additional information has been found for this compound.

**Figure 2:**
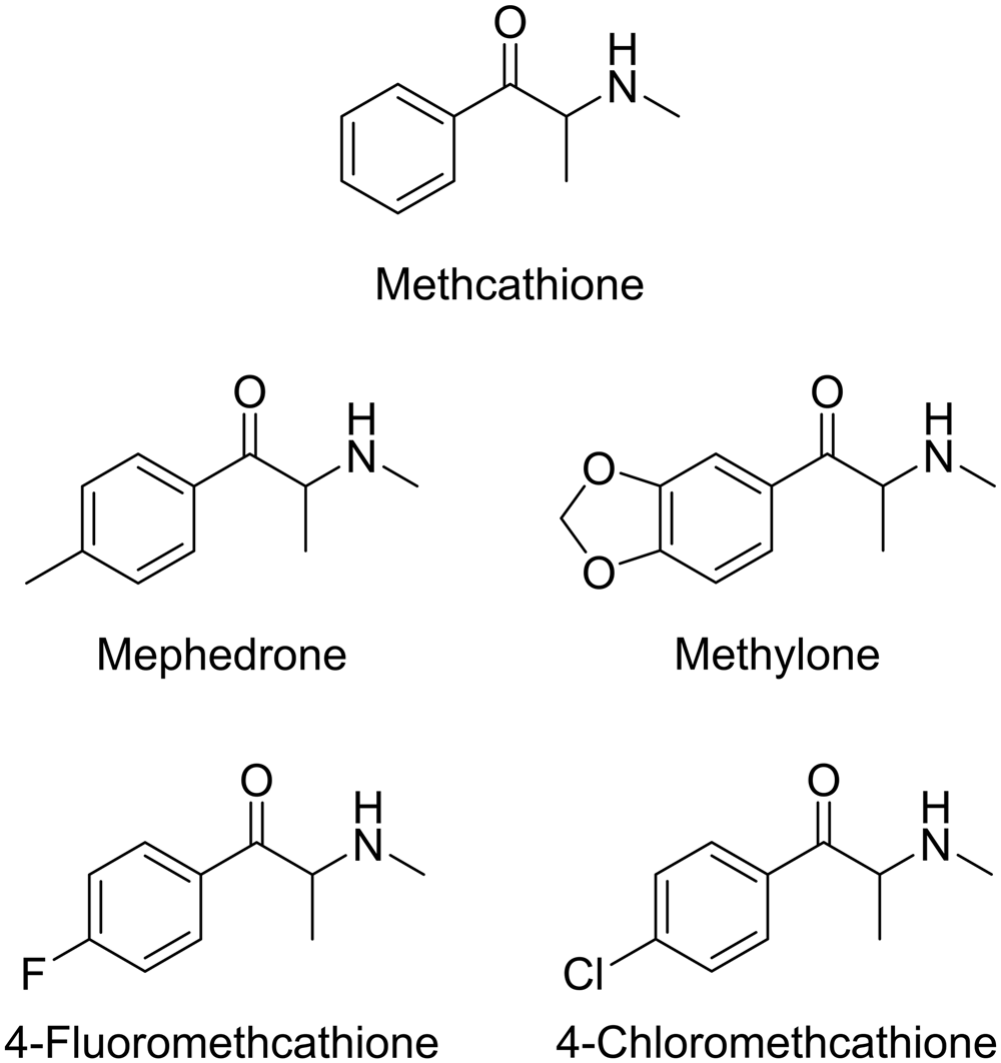
Structure of methcathinone and its reported derivatives.

The development of aminorex derivatives by modification of the aromatic ring has probably been carried out based on the “successful” synthesis of novel synthetic cathinones that use the same methodology. Two of the reported derivatives presented a *para*-methyl (4,4′-DMAR) and a 3,4-dioxane (MDMAR) moieties. These substitutions can be easily found in well-known synthetic cathinones: using the structure of methcathinone^32^, mephedrone (4-methyl-methcathinone or 4-MMC)^33^ was synthetized by adding a *para*-methyl moiety, and methylone (3,4-methylenedioxy-*N*-methylcathinone or bk-MDMA)^34^ by adding a 3,4-dioxane moiety (Fig. 2). Moreover, 4-FMC (4-fluoromethcathinone or flephedrone)^35^ and the recently reported 4-CMC (4-chloromethcathinone or clephedrone)^36^, were also synthetized based on different aromatic substitutions of methcathinone (Fig. 2). In this case, halogen atoms were used as *para*-substituents of the aromatic ring, producing novel active stimulants. In the light of this information, it would not be surprising that, in the next years, *para*-methoxy or *para*-chloro 4-MAR derivatives will appear in the streets.

Recently, a new halogenated 4-MAR derivative was detected by the Slovenian Police^37^, and reported in literature^38^. In the present study, *para*-fluoro-4-methylaminorex was fully characterized in a sample obtained from an anonymous Polish consumer. The compound was named as 4′F-4-MAR (or pF-4-methylaminorex), following a similar nomenclature to that used for other aminorex derivatives. Its characterization was performed by ultra-high performance liquid chromatography-high resolution mass spectrometry (UHPLC-HRMS) and two-dimension nuclear magnetic resonance (NMR) experiments. Analyses with gas chromatography coupled to mass spectrometry with electron ionization (GC-MS), Fourier-transformed infrared spectroscopy (FTIR), as well as ^1^H and ^13^C NMR experiments were also performed in order to provide as much information as possible. Additionally, this research illustrates how the same moiety changes applied for the preparation of synthetic cathinones are being used to prepare new stimulants based on the 4-MAR structure. All analytical information provided in this work will be useful for the identification of 4′F-4-MAR or for future 4-MAR derivatives in forensic laboratories.

## Results

### Identifying the 4′F-4-MAR

**Figure 3:**
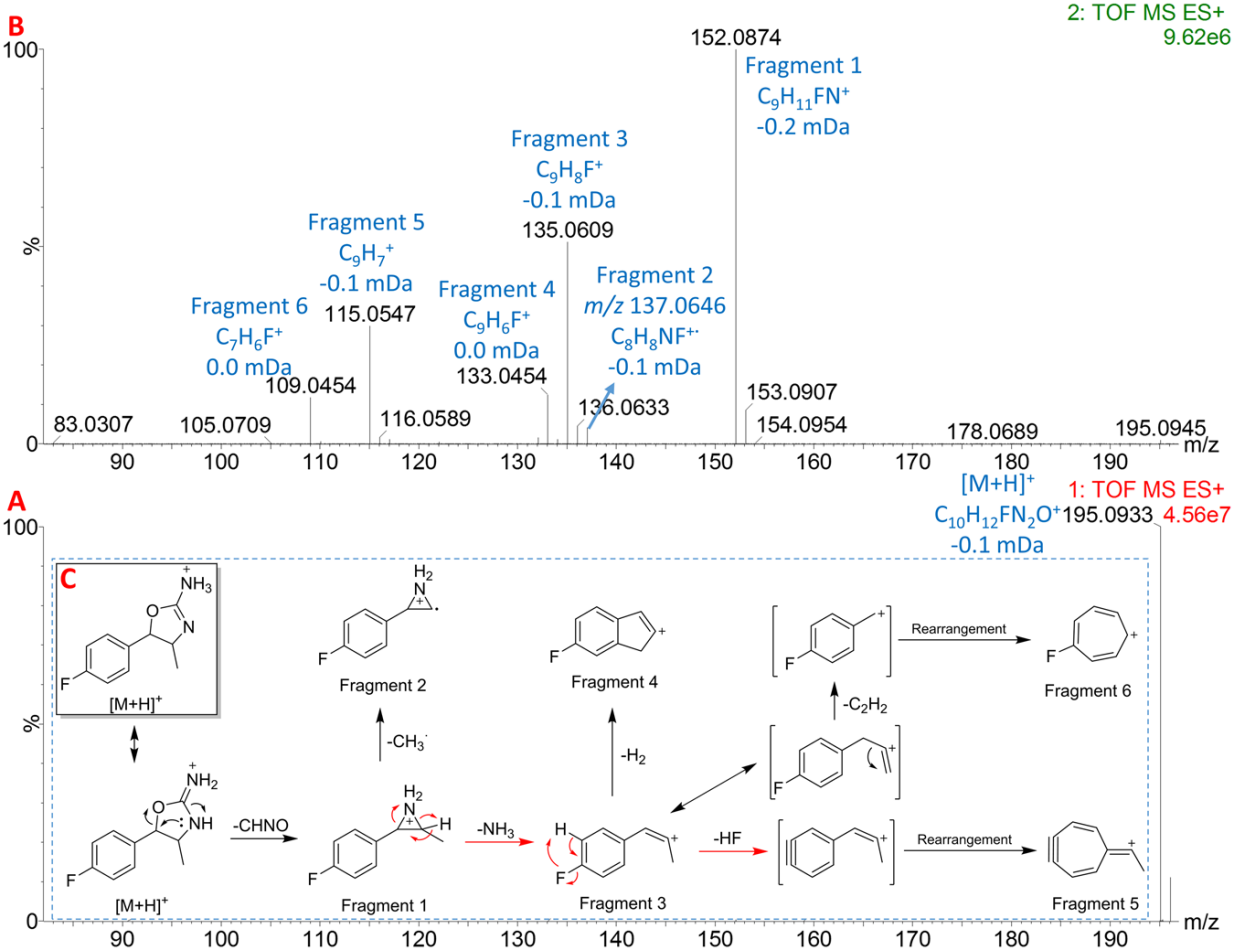
UHPLC-HRMS information of the putative aminorex derivative. (**A**) Low energy spectrum. (**B**) High energy spectrum. (**C**) Proposed fragmentation pathway once identified the new aminorex derivative.

When the compound was injected into the UHPLC-HRMS, a single chromatographic peak at 2.63 min was observed in the total ion chromatogram. Figure 3 shows the low energy and high energy spectra obtained by HRMS for this chromatographic peak. The low energy spectrum corresponding to this peak showed an ion at *m/z* 195.0933, which would correspond to the protonated molecule of the compound ([M + H]^+^) (Fig. 3A). The elemental composition for [M + H]^+^ was determined to be C_10_H_12_FN_2_O^+^ (−0.1 mDa) based on the accurate-mass observed. According to the aminorex elemental composition (C_9_H_10_N_2_O)^31^, this putative derivative should contain an extra methyl group and a fluorine atom on the aminorex structure. The high energy spectrum of the chromatographic peak showed six important fragment ions (Fig. 3B). Fragment 1 (C_9_H_11_FN^+^, −0.2 mDa) corresponds to a CHNO loss. This specific loss has been reported in literature for two aminorex derivatives, the *para*-methyl-4-methylaminorex (4,4′-DMAR) and the 3′,4′-methylenedioxy-4-methylaminorex (MDMAR)^15,31^. The loss of CHNO from [M + H]^+^ produces the formation of an *N*-epoxide structure on the molecule, in the same way than 4,4′-DMAR and MDMAR^15,31^. Fragment 3 (C_9_H_8_F^+^, −0.1 mDa) would derive from Fragment 1 after an ammonia molecule loss, according to its elemental composition. Fragment 5 (C_9_H_7_^+^, −0.1 mDa) produced after a HF molecule loss from Fragment 3, did not provide any information about the position of the fluorine atom or of the methyl group. Nevertheless, Fragment 6 (C_7_H_6_F^+^, 0.0 mDa) would correspond to a fluorotropylium ion. This fragment ion is in concordance with the tropylium ion observed for the 4,4′-DMAR^15^. Fragment 6 would indicate that the fluorine atom could be on the aromatic ring. Nevertheless, the accurate-mass fragmentation observed was not enough for confirming the identity of the compound.

**Figure 4:**
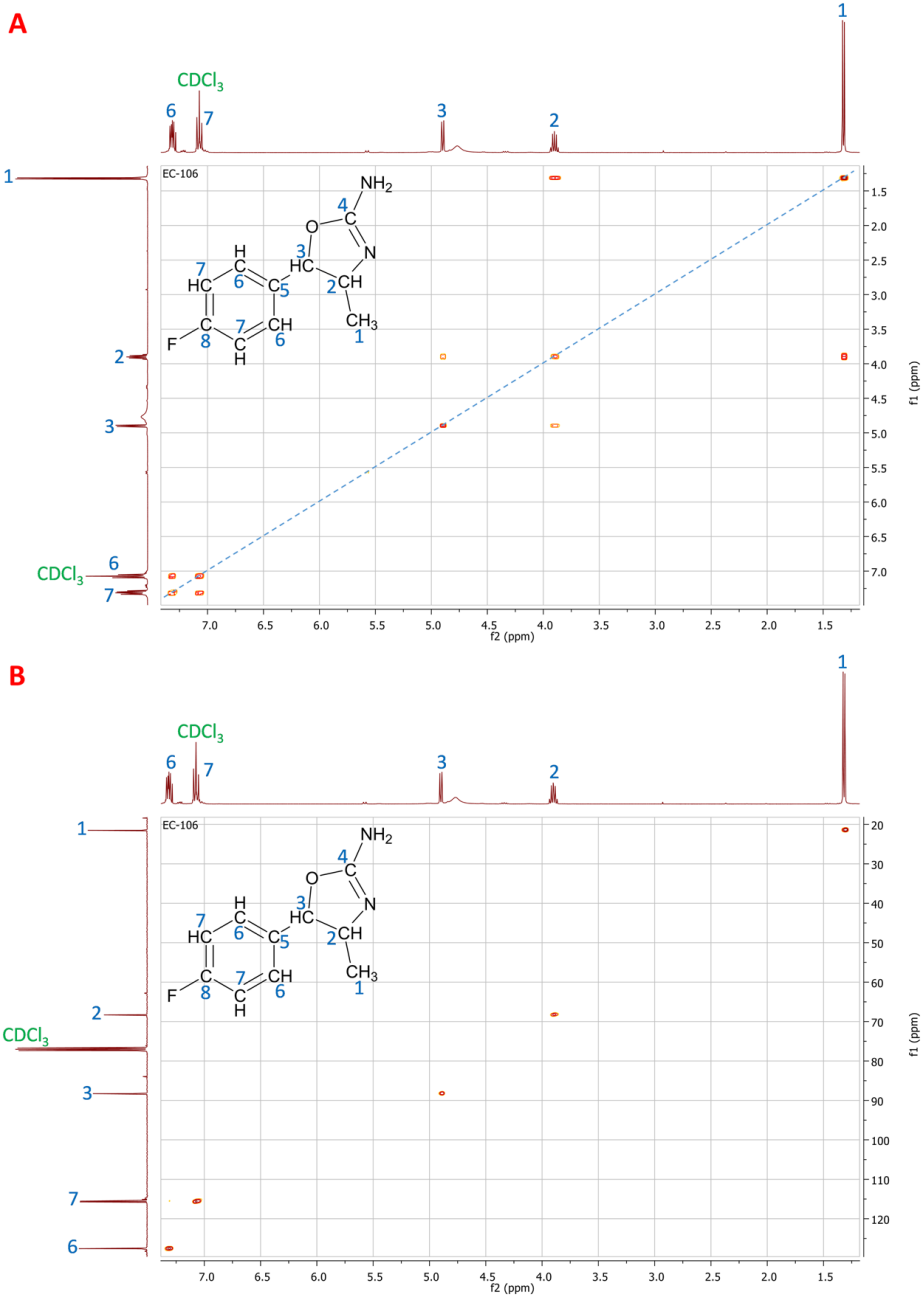
Two-dimension NMR experiments, with signal assignation, performed for the identification of the novel aminorex derivative. (**A**) COSY spectrum. (**B**) HSCQ spectrum.

In order to find the location of the methyl group and establish the position of the fluorine atom in the aromatic ring, two two-dimension NMR experiments (COSY and HSQC) were performed. Figure 4 shows the COSY spectrum (A) and the HSQC spectrum (B) of the putative aminorex derivative. The doublet at δ = 1.32 ppm, which integration was 3 in the COSY spectrum (Fig. 4A, and thus in a ^1^H spectrum) indicated the presence of a methyl group in the structure, as supposed based on the elemental composition (C_10_H_11_FN_2_O) determined by HRMS. The HSQC spectrum established that the carbon atom of this methyl was at δ = 21.50 ppm in the ^13^C spectrum (Fig. 4B), as expected for this moiety. According to the couplings observed in the COSY, this methyl group is near to a tertiary carbon (multiplet at δ = 3.90 ppm in ^1^H spectrum -Fig. 4A**-** which integrates for 1, and δ = 68.31 ppm in ^13^C -Fig. 4B-). Moreover, this tertiary atom is coupled to another tertiary atom (doublet at δ = 4.90 ppm in ^1^H spectrum -Fig. 4A**-** which integrates for 1, and δ = 88.28 ppm in ^13^C -Fig. 4B-), which multiplicity indicates that it has only one hydrogen to be coupled (the first tertiary carbon mentioned). The high δ observed for this second tertiary atom indicates that it should be near to electronegative moieties such as oxygen atoms, nitrogen atoms or aromatic rings. These observations established the methyl group to be on the position 4 of the 4,5-dihydrooxazol ring, similarly to 4-MAR^22^, 4,4′-DMAR^15,31^, and MDMAR^15,31^. Moreover, this position was in concordance with the CHNO loss observed in HRMS (Fragment 1 in Fig. 3), which can only occur if the methyl group is in this position. Once the position of the methyl group was established, the position of the fluorine atom on the aromatic ring was determined. The aromatic signals observed in the COSY spectrum (signals at δ = 7.07 and 7.31 in the ^1^H spectrum) presented the typical pattern of a *para*-substituted aromatic ring. Based on this fact and considering the fluorotropylium ion observed in HRMS (Fragment 6 in Fig. 3), the fluorine atom should be in position 4 of the aromatic ring. With this information, the novel aminorex derivative should be the *para*-fluoro-4-methylaminorex, which could be known as 4′F-4-MAR based on the nomenclature used for the other aminorex derivatives reported previously.

Once the structure of this compound was established, all the NMR signals observed in the COSY and HSQC (and thus the ^1^H and ^13^C signals) were successfully assigned to the proposed structure. Figure 4A,B show the signal assignation based on the structure of 4′F-4-MAR. The fragmentation pathway of 4′F-4-MAR was also proposed based on the accurate-mass fragmentation (Fig. 3C).

In order to provide a complete characterization of the compound and facilitate the identification of 4′F-4-MAR by forensic laboratories, GC-EI-MS and FTIR analyses were also carried out. The EI spectrum and FTIR spectrum can be found in Supplementary Information (Figs S1 and S2, respectively). The ^1^H and ^13^C NMR spectra are also provided (Figs S3 and S4, respectively). Additional information about sample treatment and instrument conditions for GC-MS and FTIR can be found in Supplementary Information.

## Discussion

A decrease in the number of new cathinones detected every year has been observed in the last few years, together with an increase in other groups of stimulants such as phenethylamines^1^. It appears that the list of possible cathinone analogues that have not been synthesized is dwindling, and producers of NPS are struggling to find substitutions that will yield an active compound^39^. Although theoretically the possibilities are nearly endless, there is a limit to the substitutions that the cathinone backbone can tolerate and still yield an active compound. It appears that the limit has nearly been reached, and producers appear to be exploring other backbones from which to create new psychoactive substances.

One example is amphetamine. In the past year, we have seen the first detection of 2-FEA, 3-FEA, and 4-FEA, three new compounds based on the amphetamine backbone^40^. These compounds follow the same modification pattern of the aromatic ring to create a new analog^41^. Nevertheless, they have reached limited popularity due to their lower effects and large required doses respect to amphetamine, as each carbon added to the amine chain of amphetamine appears to make the compound less potent^42^.

The aminoindane family has also been explored with the same pattern of limited success, with compounds such as MDAI, 5-IAI, or 2-AI being reported^43^. The same can be observed with the phenmetrazine (e.g. 3-FPM)^13^.These seem to yield compounds that are either not potent or popular enough to make their production profitable. The logical next step by producers is to continue exploring possible new families from which to create new analogues.

There are three distinct advantages that likely motivated the decision of synthesizing another aminorex analog, specifically 4′F-4-MAR: ease of synthesis, available data on effects, and users appeal.

There are discussions on synthesis forums, dating to around 2003, where a functioning synthesis is discussed and perfected^44^. For that synthesis, the described precursor is the same one used in 4-FA synthesis^45^, which means that the precursor for an NPS that is banned in several countries can be repurposed to produce a new substance not currently under strict restrictions.

Additionally, as can be seen in the archived hive thread referenced above about the synthesis of 4′F-4-MAR, bioassays by the forum users are positive, describing pleasant effects at fairly low doses and no immediate negative side effects^38^. This is very useful for producers because it removes some of the uncertainty regarding the recreational potential of this substance. Often there is no available data about effects of a new NPS on humans, and favourable *in vitro* and/or animal studies do not translate well to human effects.

The Aminorex family appears to be well-liked by forum users, as it is not explicitly mentioned in existing analog laws like the German NpSG^46^ and because of an expectation of recreational potential stemming from the effects and popularity of 4-Methylaminorex before it was banned^47^. Consequently, users will likely be willing to sample 4′F-4-MAR, and not much marketing will surely be needed by producers or vendors.

As final note, an unknown sample suspected to contain anaminorex derivative has been analysed by HRMS, allowing the tentative identification of the recently reported *para*-fluoro-4-methylaminorex or 4′F-4-MAR. The unequivocal identification and structure confirmation was performed by two-dimension NMR experiments (COSY and HSQC). The compound was also analysed by GC-EI-MS, FTIR, ^1^H and ^13^C NMR in order to provide additional information to forensic laboratories.

Based in the glorification of the effects of 4,4′-DMAR and 4-MAR by users on online forums after they were banned, it is reasonable to think that the aminorex family is an attractive candidate for the new stimulant backbone that producers are looking for^47–50^. 4′F-4-MAR appears to be the next analog that has been released. If the users evaluate this compound positively in forum discussions and demand is high, it is likely that producers will manufacture further analogues. As such, it is possible that the number of stimulants based on the cathinone backbone reported every year will keep decreasing while the number of stimulants based on aminorex backbone will continue to rise.

Data available on aminorex analogues from forums, where advanced pharmacology and synthesis are discussed, allows for a rare glimpse of the possible factors that influence the process of NPS production and distribution. Our findings support the theory that this process is relatively complex and involves a more-than-rudimentary grasp on chemistry, pharmacology, and business concepts by producers of these novel psychoactive substances.

The information provided in this paper will be of help to analytical/forensic laboratories for the identification of 4′F-4-MAR or for potential 4-MAR derivatives that may appear in the near future.

## Methods

### Research chemical sample

The research chemical sample was deferred by an anonymous user to Energy Control’s^51^ for its analysis. This sample was suspected to be a novel aminorex derivative based on the information provided by the anonymous user, so this was the premise in order to identify it.

### Reagents and chemicals

HPLC-grade water was obtained from a Milli-Q system (Millipore, Bedford, MA, USA). HPLC-grade methanol (MeOH) and acetonitrile (ACN), acetone, formic acid (HCOOH) and sodium hydroxide (NaOH) were acquired from Scharlau (Scharlab, Barcelona, Spain). Leucine enkephalin was bought from Sigma-Aldrich (St. Louis, MO, USA). Deuterated chloroform (CDCl_3_) was purchased from Sigma-Aldrich.

### Sample treatment

10 mg of sample were extracted with 1 mL of acetone in an ultrasonic bath for 15 min. After centrifugation, the supernatant was ten thousand-fold diluted with HPLC-grade water, and 20 µL of the extract were injected in the UHPLC-HRMS system.

For NMR analysis, approximately 15 mg of sample were dissolved in 0.6 mL of CDCl_3_.

### Instrumentation

Analysis was performed using an ACQUITY UPLC system (Waters, Mildford, MA, USA) coupled to a XEVO G2 QTOF mass spectrometer (Waters Micromass, Manchester, UK).A CORTECS C18 (Waters) analytical column (100 × 2.1 mm,2.7 µm particle size) at a flow rate of 0.3 mL/min was used. The column temperature was set to 40 °C. The mobile phases used were H_2_O and MeOH, both with 0.01% HCOOH. The initial percentage of organic mobile phase was changed as follows: 10% at 0 min, 90% at 14 min linearly increased, 90% at 16 min, and return to initial conditions at 18 min. The injection volume was 20 µL. Nitrogen (Praxair) was used as desolvation (1000 L/h) and nebulizing gas. TOF resolution was ~20000 at FWHM (at *m/z* 556). A mass range from *m/z* 50 to 1000 was acquired. The capillary and cone voltage were set to 0.7 kV and 20 V, respectively. Argon 99.995% (Praxair) was used as a collision gas. The interface temperature was set to 650 °C and the source temperature to 120 °C.

In MS^E^ experiments, the low energy function (LE) used a collision energy of 4 eV while the high energy function (HE) applied a collision energy ramp from 15 to 40 eV. In this way, information about the protonated molecule and adducts (if present) and fragment ions^48^ were obtained in a single analysis.

Calibration of the mass-axis was daily performed using a mixture of 0.05 M NaOH:5% HCOOH (50:50), 25-fold diluted with ACN:H_2_O (80:20). A 2 µg/mL leucine enkephalin solution in ACN:H_2_O (50:50) with 0.1% HCOOH was used as lock-mass UHPLC-HRMS data were acquired in continuum mode (MassLynx, version 4.1, Waters) and processed with UNIFI (version 1.8, Waters).

A Bruker Ascend 400 MHz spectrometer equipped with a SampleCase autosampler (Bruker, Etlingen, Germany) was used for NMR analysis. Data acquisition was performed at 303 K using CDCl_3_. The residual solvents signals at δ = 7.24 ppm for ^1^H (CHCl_3_) and at δ = 77.23 ppm for ^13^C (CDCl_3_) were used as internal references. Characterization of the compound was performed using ^1^H NMR, ^13^C NMR, correlated spectroscopy (COSY) and heteronuclear single quantum coherence (HSQC). NMR experiment data were collected using the Bruker Icon NMR 5.0.5 software (Bruker). MestreNova program was used for raw data processing (Mestrelab Research, Santiago de Compostela, Spain).

## Supplementary information

Supplementary Information

## References

[1] European Monitoring Centre for Drugs and Drug Addiction. European Drug Report 2017. EMCDDA–Europol Jt. Publ. **88**, 10.2810/610791 (2017).

[2] Fornal E (2013). Identification of substituted cathinones: 3,4-Methylenedioxy derivatives by high performance liquid chromatography–quadrupole time of flight mass spectrometry. J. Pharm. Biomed. Anal..

[3] Qian Z, Jia W, Li T, Liu C, Hua Z (2017). Identification and analytical characterization of four synthetic cathinone derivatives iso-4-BMC, β -TH-naphyrone, mexedrone, and 4-MDMC. Drug Test. Anal..

[4] Liu C, Jia W, Li T, Hua Z, Qian Z (2017). Identification and analytical characterization of nine synthetic cathinone derivatives N -ethylhexedrone, 4-Cl-pentedrone, 4-Cl- α -EAPP, propylone, N -ethylnorpentylone, 6-MeO-bk-MDMA, α -PiHP, 4-Cl- α -PHP, and 4-F- α -PHP. Drug Test. Anal..

[5] Brandt SD, Daley PF, Cozzi NV (2012). Analytical characterization of three trifluoromethyl-substituted methcathinone isomers. Drug Test. Anal..

[6] Doi T (2016). Identification and characterization of a-PVT, a-PBT, and their bromothienyl analogs found in illicit drug products. Forensic Toxicol..

[7] Apirakkan Orapan, Frinculescu Anca, Shine Trevor, Parkin Mark C., Cilibrizzi Agostino, Frascione Nunzianda, Abbate Vincenzo (2017). Analytical characterization of three cathinone derivatives, 4-MPD, 4F-PHP and bk-EPDP, purchased as bulk powder from online vendors. Drug Testing and Analysis.

[8] Gaspar H (2015). 4F-PBP (4′-fluoro-α-pyrrolidinobutyrophenone), a new substance of abuse: Structural characterization and purity NMR profiling. Forensic Sci. Int..

[9] Fabregat-Safont D (2018). Reporting the novel synthetic cathinone 5-PPDI through its analytical characterization by mass spectrometry and nuclear magnetic resonance. Forensic Toxicol..

[10] Assi S, Gulyamova N, Kneller P, Osselton D (2017). The effects and toxicity of cathinones from the users’ perspectives: A qualitative study. Hum. Psychopharmacol. Clin. Exp..

[11] European Monitoring Centre for Drugs and Drug Addiction. European Drug Report 2018. EMCDDA Publ, 10.2810/88175 (2018).

[12] Johansen SS, Hansen TM (2012). Isomers of fluoroamphetamines detected in forensic cases in Denmark. Int. J. Legal Med..

[13] McLaughlin G (2017). Test purchase, synthesis and characterization of 3-fluorophenmetrazine (3-FPM) and differentiation from its ortho - and para -substituted isomers. Drug Test. Anal..

[14] Davidson C, Raby CAR, Barrese V, Ramsey J (2018). *In Vitro* Neurochemical Assessment of Methylphenidate and Its “Legal High” Analogs 3,4-CTMP and Ethylphenidate in Rat Nucleus Accumbens and Bed Nucleus of the Stria Terminalis. Front. Psychiatry.

[15] Brandt SD (2014). Characterization of a novel and potentially lethal designer drug (±)-cis-para-methyl-4-methylaminorex (4,4′-DMAR, or ‘Serotoni’). Drug Test. Anal..

[16] McNeil Laboratories. 2-AMINO-5-ARYLOXAZOLINE PRODUCTS (US Patent Office 3,161,650). *US Pat. Off*. (1964).

[17] Weigle DS (2003). Pharmacological Therapy of Obesity: Past, Present, and Future. J. Clin. Endocrinol. Metab..

[18] Gaine SP, Rubin LJ, Kmetzo JJ, Palevsky HI, Traill TA (2000). Recreational Use of Aminorex and Pulmonary Hypertension. Chest.

[19] Fishman AP (1999). Aminorex to Fen/Phen: An Epidemic Foretold. Circulation.

[20] Drug Enforcement Administration. Lists of: Scheduling Actions, Controlled Substances, Regulated Chemicals. *U*.*S*. *Dep*. *Justice* (2018).

[21] McNeil Laboratories. 2-AMINO-5-ARYLOXAZOLINE COMPOSITIONS AND METHODS OF USING SAME (US Patent Office 3,278,382). *US Pat. Off*. (1966).

[22] Young R, Glennon RA (1993). Cocaine-stimulus generalization to two new designer drugs: Methcathinone and 4-methylaminorex. Pharmacol. Biochem. Behav..

[23] Russell BR, Beresford RA, Schmierer DM, McNaughton N, Clark CR (1995). Stimulus properties of some analogues of 4-methylaminorex. Pharmacol. Biochem. Behav..

[24] Glennon RA, Misenheimer B (1990). Stimulus properties of a new designer drug: 4-methylaminorex (“U4Euh”). Pharmacol. Biochem. Behav..

[25] Bunker CF, Johnson M, Gibb JW, Bush LG, Hanson GR (1990). Neurochemical effects of an acute treatment with 4-methylaminorex: a new stimulant of abuse. Eur. J. Pharmacol..

[26] Davis FT, Brewster ME (1988). A fatality involving U4Euh, a cyclic derivative of phenylpropanolamine. J. Forensic Sci..

[27] Maier J (2018). The psychostimulant (±)-cis-4,4′-dimethylaminorex (4,4′-DMAR) interacts with human plasmalemmal and vesicular monoamine transporters. Neuropharmacology.

[28] EMCDDA-Europol. Link suspected between deaths and new psychoactive substance: 4-methylaminorex, para-methyl derivative. EARLY Warn. Notif. (2014).

[29] EMCDDA-Europol. EMCDDA–Europol Joint Report on a new psychoactive substance: 4,4′-DMAR (4-methyl-5-(4-methylphenyl)-4,5-dihydrooxazol-2-amine). EMCDDA–Europol Jt. Publ (2014).

[30] European Union. COUNCIL IMPLEMENTING DECISION (EU) 2015/1873. *Off. J. Eur. Union* 32–34 (2015).

[31] McLaughlin G (2015). Synthesis, characterization, and monoamine transporter activity of the new psychoactive substance 3′,4′-methylenedioxy-4-methylaminorex (MDMAR). Drug Test. Anal..

[32] Calkins RF, Aktan GB, Hussain KL (1995). Methcathinone: The Next Illicit Stimulant Epidemic?. J. Psychoactive Drugs.

[33] Winstock AR (2011). Mephedrone, new kid for the chop?. Addiction.

[34] Bossong MG, Van Dijk JP, Niesink RJM (2005). Methylone and mCPP, two new drugs of abuse?. Addict. Biol..

[35] Archer RP (2009). Fluoromethcathinone, a new substance of abuse. Forensic Sci. Int..

[36] Taschwer M, Weiß JA, Kunert O, Schmid MG (2014). Analysis and characterization of the novel psychoactive drug 4-chloromethcathinone (clephedrone). Forensic Sci. Int..

[37] National Forensic Laboratory of Slovenia. *ANALYTICAL REPORT*. *pF-4-methylaminorex* (*C10H11FN2O*). *European Project RESPONSE 2*.

[38] Maier J, Mayer FP, Brandt SD, Sitte HH (2018). DARK Classics in Chemical Neuroscience: Aminorex Analogues. ACS Chem. Neurosci..

[39] Fabregat-Safont D (2017). Updating the list of known opioids through identification and characterization of the new opioid derivative 3,4-dichloro-N-(2-(diethylamino)cyclohexyl)-N-methylbenzamide (U-49900). Sci. Rep..

[40] Yanini Á, Armenta S, Esteve-Turrillas FA, Galipienso N, de la Guardia M (2018). Identification and characterization of the new psychoactive substance 3-fluoroethamphetamine in seized material. Forensic Toxicol..

[41] Grifell M (2017). Patterns of use and toxicity of new para-halogenated substituted cathinones: 4-CMC (clephedrone), 4-CEC (4-chloroethcatinone) and 4-BMC (brephedrone). Hum. Psychopharmacol. Clin. Exp..

[42] 3-fea. bluelight.org (2017). Available at, http://bluelight.org/vb/threads/813802-3-fea/page2. (Accessed: 24th June 2018).

[43] Pinterova, N., Horsley, R. R. & Palenicek, T. Synthetic Aminoindanes: A Summary of Existing Knowledge. *Front*. *Psychiatry* **8** (2017). 10.3389/fpsyt.2017.00236 PMC5698283 29204127

[44] Synthesis of para-fluoro-(4-methylaminorex). chemistry.mdma.ch Available at. https://chemistry.mdma.ch/hiveboard/methods/000464621.html. (Accessed: 24th June 2018).

[45] 4-Fluoroamphetamine Synthesis. erowid.org Available at, https://erowid.org/archive/rhodium/chemistry/pfa.spicybrown.html. (Accessed: 24th June 2018).

[46] Bundesgesetzblatt Jahrgang. Gesetz zur Bekämpfung der Verbreitung neuer psychoaktiver Stoffe. **2016**, 2615–2622 (2016).

[47] 2′-Fluoro-4-Methylaminorex. He’s done it. reddit.com (2018). Available at, https://www.reddit.com/r/researchchemicals/comments/7xuj76/2fluoro4methylaminorex_hes_done_it/. (Accessed: 24th June 2018).

[48] Euphoria of the Dick. 4-Methylaminorex. erowid.org (2018). Available at, https://erowid.org/experiences/exp.php?ID=106177. (Accessed: 2nd May 2019).

[49] 4-MAR (U4EA). sixthseal.com (2003). Available at, http://sixthseal.com/2003/04/4-mar-u4ea/. (Accessed: 2nd May 2019).

[50] The synthesis of 2′Fluoro-4-Methylaminorex. reddit.com (2018). Available at, https://www.reddit.com/r/TheeHive/comments/7xvd5d/the_synthesis_of_2fluoro4methylaminorex/. (Accessed: 2nd May 2019).

[51] González D, Ventura M, Caudevilla F, Torrens M, Farre M (2013). Consumption of new psychoactive substances in a Spanish sample of research chemical users. Hum. Psychopharmacol Clin Exp.

