# Supplementary material for: Characterization of a recently detected halogenated aminorex derivative: para-fluoro-4-methylaminorex (4′F-4-MAR)
Source: Sci Rep. 2019 Jun 5;9:8314. doi: 10.1038/s41598-019-44830-y (PMC6549166; doi:10.1038/s41598-019-44830-y)
Supplement: Supplementary file 1 — Supplementary Information [file 41598_2019_44830_MOESM1_ESM.pdf]

Supplementary Information

Characterization of a recently detected halogenated aminorex derivative: *para*-fluoro-4-methylaminorex (4'F-4-MAR).

D. Fabregat-Safont[1], X. Carbón[2], M. Ventura[2], I. Fornís[2], F. Hernández[1], M. Ibáñez[1]

[1] Research Institute for Pesticides and Water, University Jaume I, Avda. Sos Baynat s/n, 12071, Castelló de la Plana, Spain.

[2] Energy Control (Asociación Bienestar y Desarrollo), c/ Independencia 384, 08041, Barcelona, Spain.

Correspondence and request for materials should be addressed to M.I. (email: ibanezm@uji.es).

Methods

Reagents and chemicals

For GC-MS analysis, GC-grade n-hexane and GC-grade acetone were purchased from Scharlau (Scharlab, Barcelona, Spain). For FTIR analysis, potassium bromide (KBr) was purchased from Scharlau.

Sample treatment

For FTIR analysis, sample was prepared with 5% of the unknown compound and 95% of potassium bromide, homogenized in an agate mortar and compressed under a pressure of 5000 kg/cm^2.

For GC-MS analysis, 10 mg of sample were extracted with 1 mL of acetone in an ultrasonic bath for 15 min. After centrifugation, the supernatant was five thousand-fold diluted with GC-grade n-hexane, and 1 μL of the extract were injected in the GC-MS system.

Instrumentation

For FTIR analysis, a Jasco FT/IR-6200 FTIR spectrometer (Jasco Inc., Easton, MD, USA) was used. Data acquisition was performed at 23 ºC between 4000 and 400 cm^{-1}, with a resolution of 4 cm^{-1} and performing 32 acquisitions.

For GC-MS analysis, an Agilent 6890N gas chromatograph (Agilent Technologies, Santa Clara, CA, USA) equipped with an Agilent 7683 autosampler (Agilent Technologies) was coupled to a Quattro Micro GC triple quadrupole mass spectrometer (Micromass, Boston, USA) using an electron ionization (EI) interface. The injector and the interface were operated at 250 ºC. 1 µL of sample was injected in splitless

mode using deactivated liners into a 30 m 0.25 mm i.d., 0.25 μm film thickness DB-5MS column (Agilent). Helium (99.999%, Praxair, Valencia, Spain) was used as carrier gas at a flow rate of 1 mL/min. The oven temperature was initially maintained at 90 ºC for 1 min and programmed to reach 300 ºC at 20 ºC per min. It was finally maintained at 300 ºC for 1.5 min (total run time was 12 min). The mass spectrometer was operated in electronic ionization mode at 70 eV. MS system worked in SCAN acquisition mode, acquiring from *m/z* 50 to 400 Da. Analytical data were acquired and processed using MassLynx data station operation software (version 4.0, Waters).

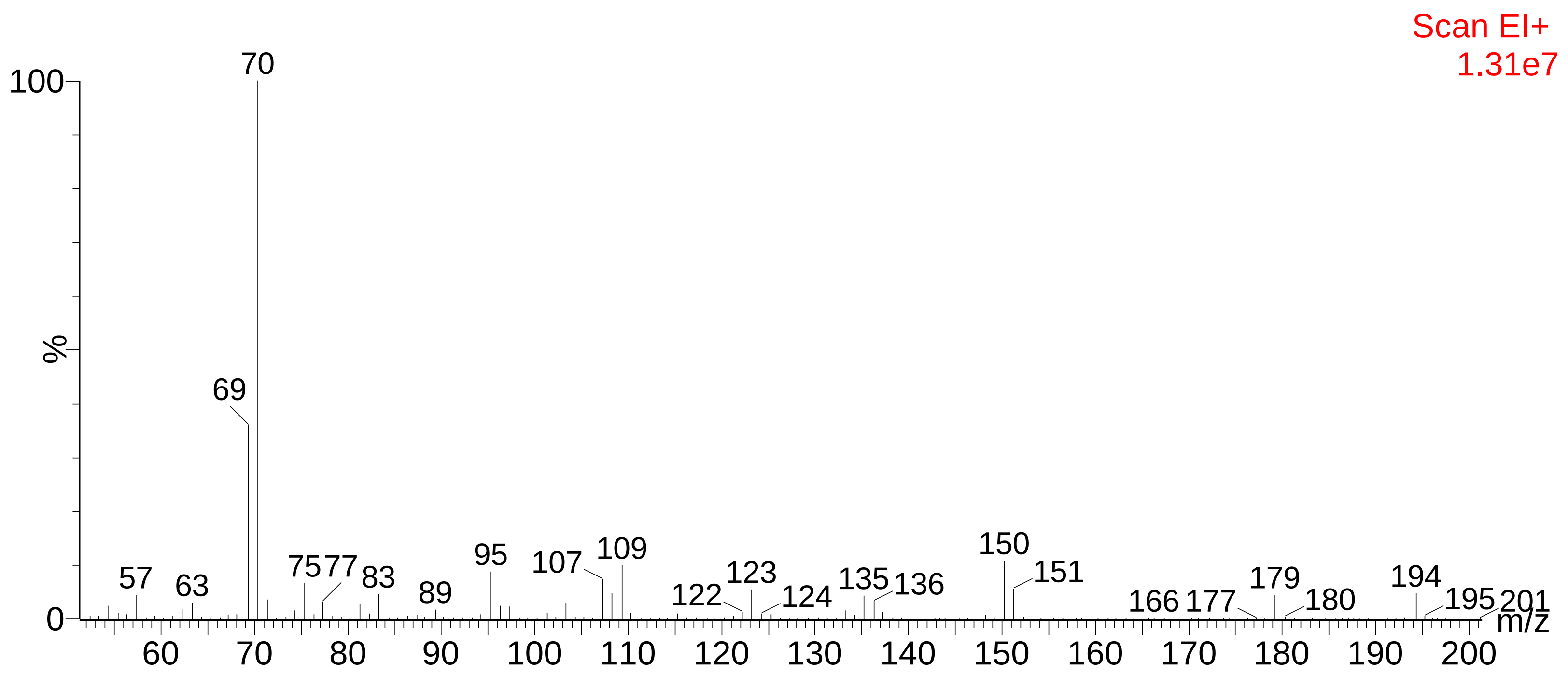

Fig. S1. GC-(EI)MS spectrum of the 4’-fluoro-4-MAR.

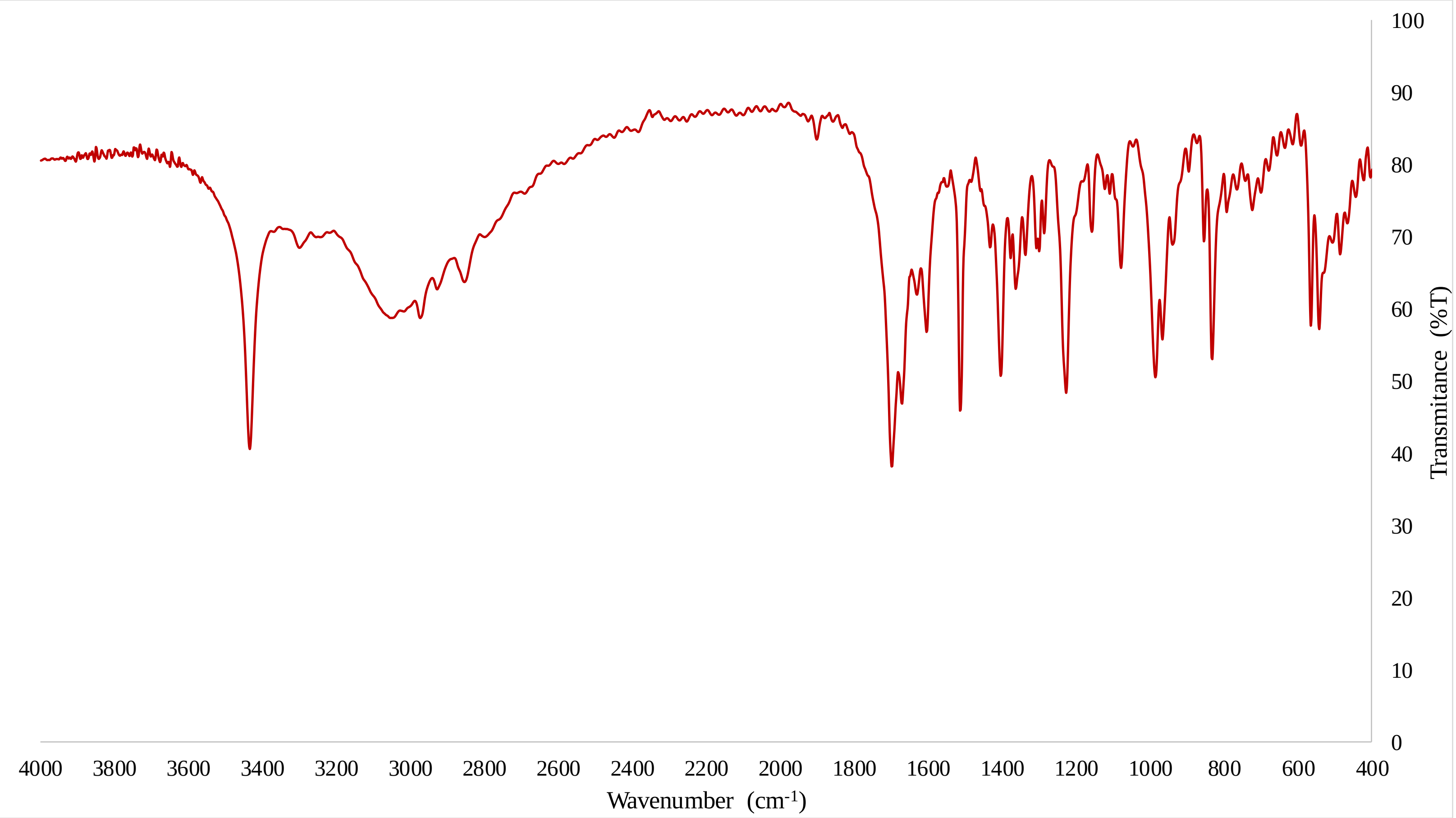

Fig. S2. FTIR spectrum of the 4'-fluoro-4-MAR.

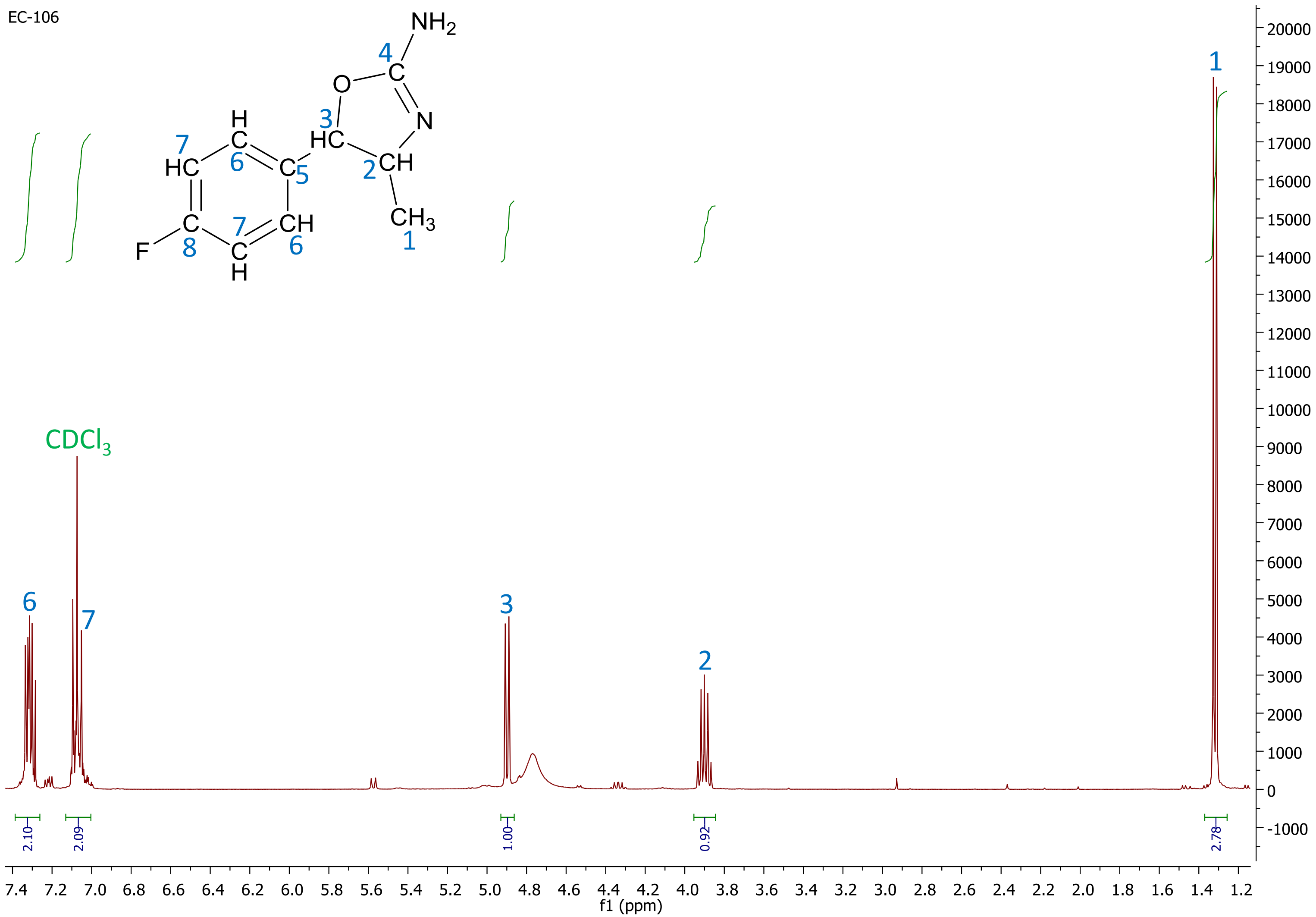

Fig. S3. ^{1}H NMR spectrum of the 4'-fluoro-4-MAR.

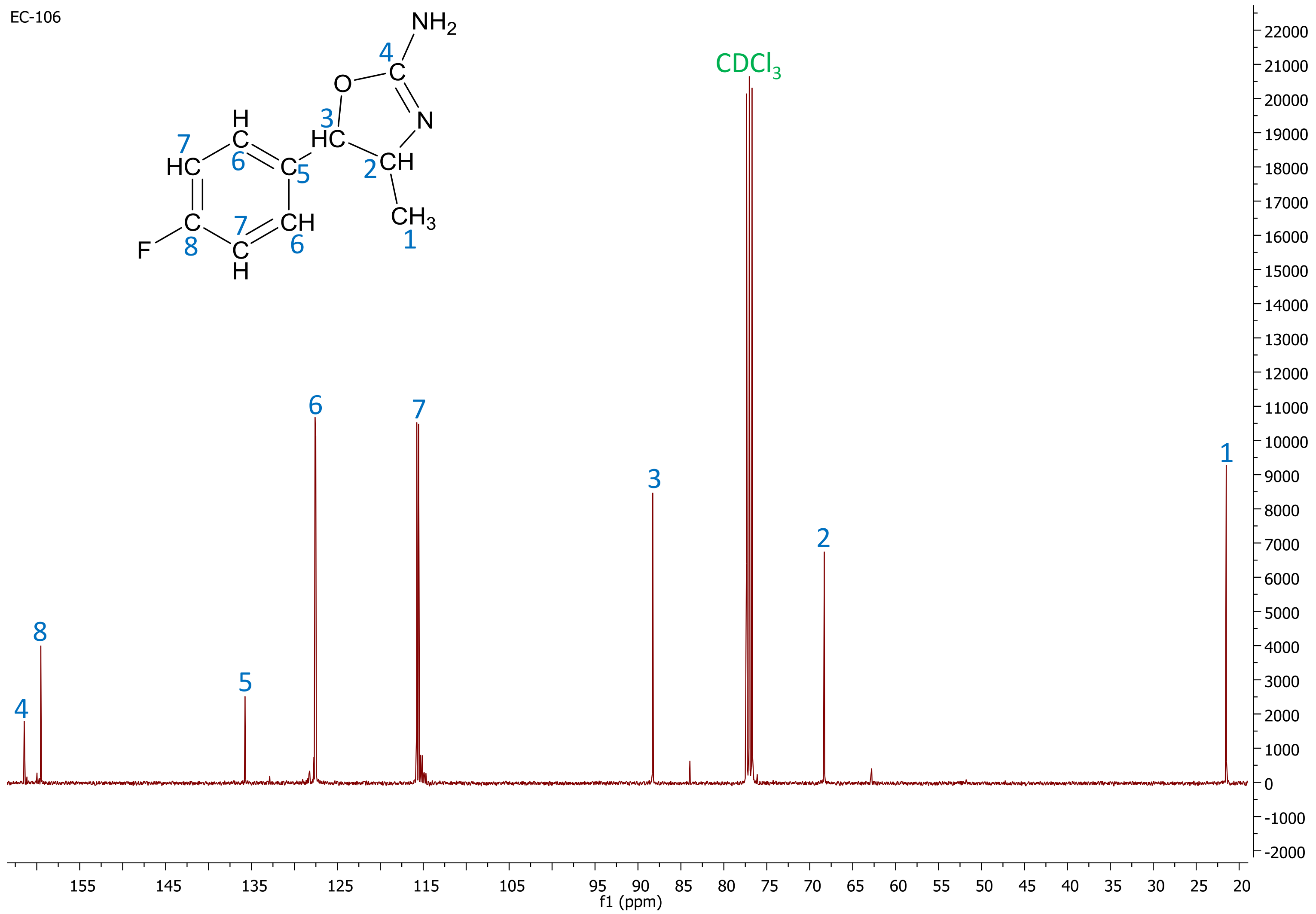

Fig. S 4. ^{13}C NMR spectrum of the 4’-fluoro-4-MAR.
